# A Review on Micro-Watts All-Digital Frequency Synthesizers

**DOI:** 10.3390/mi16030333

**Published:** 2025-03-13

**Authors:** Venkadasamy Navaneethan, Boon Chiat Terence Teo, Annamalai Arasu Muthukumaraswamy, Xian Yang Lim, Liter Siek

**Affiliations:** 1 School of Electrical and Electronic Engineering, Nanyang Technological University, Singapore 639798, Singaporee190014@e.ntu.edu.sg (X.Y.L.); elsiek@ntu.edu.sg (L.S.); 2 CM Engineering Labs Singapore Pte. Ltd., Singapore 608526, Singapore; annamalai.muthu@cmelabs.com.sg; 3 Silicon Laboratories International Pte. Ltd., Singapore 539775, Singapore; 4 Analog Devices Singapore, Singapore 349248, Singapore

**Keywords:** phase-locked loop (PLL), time-to-digital converter (TDC), sub-sampling PLL (SS-PLL), oversampling PLL (OS-PLL), sigma-delta-modulator (SDM), counter-assisted ADPLL, divider-based ADPLL

## Abstract

This paper reviews recent developments in highly integrated all-digital frequency synthesizers suitable to deploy in low-power internet-of-things (IoT) applications. This review sets low power consumption as a key criterion for exploring the all-digital frequency synthesizer implemented in CMOS fabrication technology. The alignment with mainstream CMOS technology offers high-density, comprehensive, robust signal processing capability, making it very suitable for all-digital phase-locked loops to harvest that capacity, and it becomes inevitable. This review includes various divider-less low-power frequency synthesizers, including all-digital phase-locked loops (ADPLL), all-digital frequency-locked loops (ADFLL), and hybrid PLLs. This paper also discusses the latest architectural developments for ADPLLs to lead to low-power implementation, such as DTC-assisted TDC, embedded TDC, and various levels of hybridization in ADPLLs.

## 1. Introduction

The trend and paradigm shift toward an autonomous system led to the development of numerous use cases. Many of the applications are built around autonomous sensing and communication. This led to greater automation and offloading of data to the computing server. Therefore, highly efficient low-power battery-operated integrated circuits (IC) are essential for radio frequency (RF) communication links and signal processing. At the same time, modern semiconductor processing technology paved the way for highly integrated complex functionalities. Also, contemporary packaging technologies allow for the integration of multi-chip solutions such as chiplets, through-silicon-via (TSV), and silicon interposers, enabling 3D integrated circuits (IC) to improve available system functionality and capacity [1,2,3,4]. This level of integration also allows us to integrate the battery into the miniaturized system, and the battery can also be augmented using energy harvesting techniques, leading to a completely self-sustainable autonomous system. Recently, there has been a sudden surge in the application space, and manufacturing technology has involuntarily steered the need for low-power RF transceivers for autonomous sensing, low-power wearables, and mobile communication. In RF chips, local oscillator (LO) generation is predominantly conducted by phase-locked loops (PLLs) and often by all-digital PLLs (ADPLL) instead of analog PLLs. The classical analog PLLs need to employ bulky loop filters, which leads to poor area utilization and less flexibility for calibration of loop bandwidth (BW), frequency sensitivity (K_vco_)_,_ and voltage-controlled oscillator (VCO) linearization. Meanwhile, the digital-intensive ADPLLs using digitally controlled oscillators (DCO) are suitable for meeting various requirements such as BW calibration, K_DCO_ (sensitivity of DCO), linearization, and adaptability.

**Figure 1:**
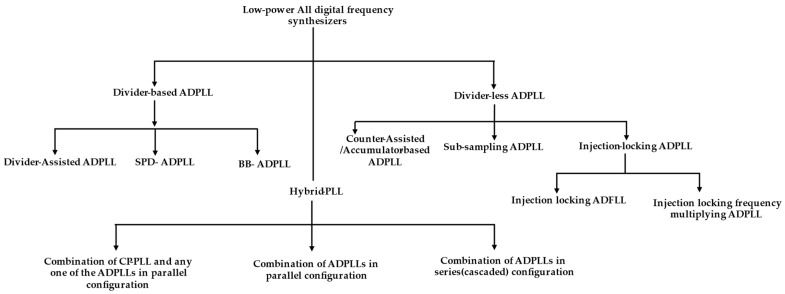
Hierarchical classification of an all-digital frequency synthesizer [7,8,9].

**Figure 2:**
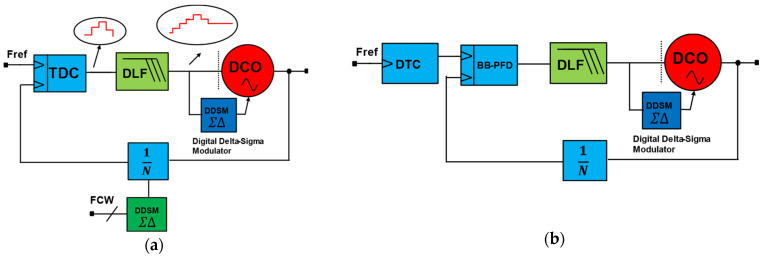
ADPLL using dividers in feedback path: (**a**) divider-assisted ADPLL; (**b**) bang-bang PFD-based ADPLL (BB-ADPLL).

At the same time, the usage of ADPLLs is ever-increasing due to the rapid design portability from one technology node to another. As shown in Figure 1, all-digital phase-locked loops can be divided into two main categories: (1) divider-based ADPLLs; and (2) divider-less ADPLLs. The divider-based ADPLLs can be divided into divider-assisted ADPLLs [5] and bang-bang phase-frequency detector (PFD)-based ADPLLs (BB-ADPLL). The divider-less ADPLLs can be further grouped into sub-sampling ADPLLs (SS-ADPLLs), accumulator-based ADPLLs [6] (counter-assisted), and injection-locked ADPLLs. There is another broader group called hybrid ADPLLs. A hybrid ADPLL can be a combination of one of the ADPLLs, a combination of ADPLLS, and can be a combination of an analog PLL and ADPLL. It can also combine two types of ADPLLs connected in parallel or cascade fashion. Numerous studies have been reported on designs for extremely low-power ADPLLs. Some implementations are based on ring-oscillator (RO) and others on inductor-capacitor (LC) oscillators. The divider-assisted ADPLLs are the direct digital equivalent of analog charge-pump (CP)-PLLs where DCO replaces VCO, and time-to-digital converter (TDC) replaces both PFD and CP. In general, SS-PLLs and BB-ADPLLs can produce integer-N frequency synthesis; however, the fractional-N operation is also demonstrated in more modern implementations with DTC assistance. Figure 2a shows the divider-assisted ADPLL. The TDC combines the operation of PFD and CP and acts as a phase detector that compares the phase or time difference of reference frequency and multi-modulus divider (MMD) output, which is essentially a divided version of DCO frequency. It produces quantized or digital code equivalent to the phase difference fed to the digital-loop filter (DLF) for type-II operation. Now, DLF produces an oscillator tuning word (OTW) to drive the DCO toward the target frequency. As we can notice, this ADPLL requires power-hungry TDC and two sigma-delta modulators (SDM) for fruitful closed-loop operation. One of the SDMs is used by DCO to produce a fine average frequency from DCO by SDM dithering and enables quantization noise shaping.

Meanwhile, the other SDM is used to dither MMD to synthesize fractional division for fractional-N operation. As this architecture necessitates the usage of a wide range of TDC and SDMs, power scaling toward the sub-milli watts regime is difficult to achieve. Nevertheless, there are some low-power implementations with narrow-range TDC with phase prediction. In the case of BB-ADPLLs, achieving high performance requires high power. Figure 2b shows BB-ADPLL, which requires MMD in its feedback path. The divided clock will be used for phase comparison by bang-bang PFD to phase error. The PFD output will go through DLF to produce a filtered version of the phase error constituting the OTW. The OTW will drive the DCO toward the target as there is a closed-loop system if sufficient stability criteria are met. Reference [10] implemented BB-ADPLL capable of synthesizing fractional-N frequency with the DTC assistance.

## 2. Review on Low-Power ADPLLs

**Figure 3:**
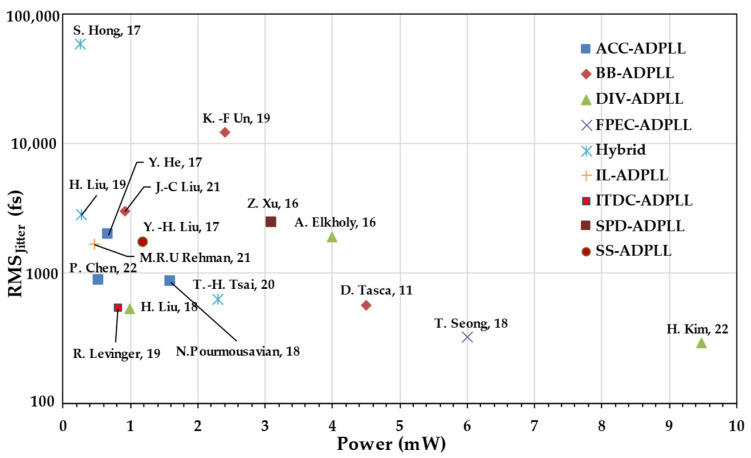
RMS Jitter of PLL versus power [8,9,10,12,13,14,15,16,17,18,19,20,21,22,23].

**Figure 4:**
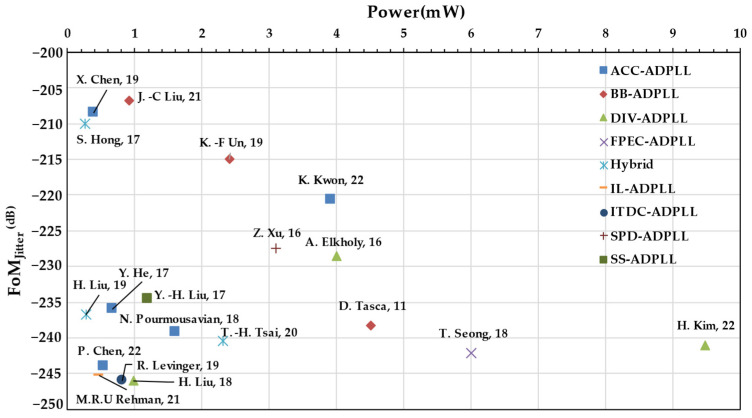
*FoM_J_* (Jitter) of PLL versus power [7,8,9,10,11,12,13,14,15,16,17,18,19,20,21,22,23,24,25].

This review mainly focuses on divider-less low-power PLLs such as accumulator/counter-based ADPLLs [11], SS-ADPLLs, ADFLLs, and hybrid ADPLLs. But before diving deep into all-digital frequency synthesizers in the divider-less category, reviewing various low-power sub-milli-watt PLLs is essential. Table 1 shows the performance summary of low-power ADPLLs. Also, this review captures the performance summary in a scatter plot for ease of visualization. Figure 3 shows a scatter plot of power versus (root-mean-square) RMS jitter, and Figure 4 shows power versus *FoM_J_*, which can be given by the following expression below [12]. Where *P* is the power consumption and σ is the variance of the integrated jitter.

(1) $$FoM_{J}=10\log\left(\left(\frac{\sigma^{2}}{1s}\right)\left(\frac{P}{1mW}\right)\right)$$

**Table 1:** Performance summary of various low-power frequency synthesizers.

No.	PLL Type	Tech	Power (mW)	* IPN-BW	Jitter (rms)	*FoM_J_* (dB)	Area in sq·mm	Key Techniques
**[12]**	ACC-ADPLL	65 nm	0.53	10 k–10 M	0.87 ps	−244	0.42	Class-F^−1^ DCO
**[13]**	ITDC-ADPLL	28 nm	0.82	10 k–20 M	543 fs	−246	0.105	^a^ ITDC-ADPLL
**[14]**	DIV-ADPLL	65 nm	0.98	10 k–10 M	530 fs	−246	0.23	TDC + DTC
**[15]**	ACC-ADPLL	40 nm	0.67	NA	1.98 ps	−236	0.18	
**[11]**	ACC-ADPLL	40 nm	0.38	-	-	−208.5	-	
**[24]**	ACC-ADPLL	12 nm	3.91	10 k–10 M	-	−220.7	0.063	
**[25]**	ACC-ADPLL	28 nm	1.6	-	0.86 ps	−239.2	0.33	
**[16]**	BB-ADPLL	65 nm	2.4	10 k–100 M	12 ps	−215	0.12	
**[10]**	BB-ADPLL	65 nm	4.5	-	560 fs	−238.3	0.22	
**[26]**	ADFLL	180 nm	1.26	-	-	-	0.14	^b^ FDC and DAC
**[17]**	BB-ADPLL	90 nm	0.912	10 k–10 M	2.99 ps	−206.8	0.007	
**[27]**	ADFLL	180 nm	1.26	-	-	-	0.14	^b^ FDC and DAC
**[18]**	DIV-ADPLL	65 nm	4	10 k–100 M	1.9 ps	−228.5	0.084	
**[28]**	CP-PLL	65 nm	0.43	-	-	-	0.54	CP-PLL
**[19]**	FPEC-ADPLL	65 nm	6	1 k–100 M	320 fs	−242.1	0.055	^c^ FPEC
**[20]**	DIV-ADPLL	40 nm	9.48	-	289 fs	−241	0.055	^e^ ACSC
**[7]**	Hybrid	65 nm	0.25	-	58 ps	−210.08	0.083	M&S IL-ADPLL
**[29]**	ACC-ADPLL	40 nm	1.4	-	-	-	-	RO IL-TDC
**[8]**	Hybrid	7 nm	2.3	-	0.619 ps	−245.5	0.012	Hybrid (ADPLL + CP)
**[30]**	AADFLL	55 nm	0.48	-	-	-	0.0798	FLL
**[21]**	IL-ADPLL	55 nm	0.46	-	1.652 ps	−245.3	0.129	IL-FLL
**[22]**	SPD-PLL	65 nm	3.1	10 k–100 M	2.4 ps	−227.5	0.064	SAR Based ^d^ SPD
**[23]**	SS-ADPLL	40 nm	1.19	NA	1.7 ps	−234.6	0.22	SS-ADPLL
**[31]**	SS-PLL	40 nm	1.03	1 k–100 M	375 fs	−253.8	0.197	LV SS-PLL(Analog)
**[9]**	SS-ADPLL	65 nm	0.265	-	2.8 ps	−236.8	0.25	Hybrid (SS/DIV ADLL)

* IPN-BW—integrated phase noise-bandwidth; ^a^ ITDC—interpolative TDC; ^b^ FDC—frequency-to-digital converter; ^c^ FPEC—fast phase error correction; ^d^ SPD—sampling phase detector; ^e^ ACSC—analog closed loop for supply noise compensation.

## 3. Divider-Less Low-Power ADPLLs

Several low-power dividers-less ADPLLs are spanning across various architectures and topologies. The subsequent sections will discuss divider-less ADPLLs aiming for low-power operation. The divider-less operation eliminates the need for MMD, and with the reduced-range DTC-assisted TDC, several low-power ADPLLs have been reported. This contrasts with the divider-based ADPLLs, which require multiple SDMs, MMD, and wide-range TDC. As a result, divider-less operation has a simpler architecture without using a wide range TDC and SDMs, power scaling toward the sub-milli watts regime. Nevertheless, there are some low-power divider-based ADPLL implementations with narrow-range TDCs. However, this review mainly focuses on a broader category of divider-less low-power PLLs, such as accumulator/counter-assisted ADPLLs, SS-ADPLLs, ADFLLs, and hybrid ADPLLs.

### 3.1. Latest Developments in Low-Power Accumulator-Based ADPLLs

Negative feedback frequency synthesizers are commonly used frequency synthesizers. The accumulator-based ADPLL is also one such frequency synthesizer that uses closed-loop negative feedback [6] and the detailed modeling and operation are given in [32]. Several improvements were made to the accumulator-based ADPLLs in the DCO and TDC along with some architecture changes to incorporate low-power capability. Classical improvements have been made in DCO power efficiency to meet high-performance phase noise. A very low-power inverse class-F DCO [12] is implemented with transformer coupling to enhance the phase noise and low voltage compliance. An enhancement in the low-power DCO buffer design exploits the voltage transfer characteristics is reported in [33]. On the architectural front, including DTC assistance reduces TDC range and operating frequency [33]. Earlier architectures used DCO frequency directly for the operation of TDC to quantize the time between DCO and REF edges. Improved techniques to extend the range and linearity of TDCs have been reported. Also, many more techniques are introduced to calibrate DTC to minimize spur. Several low-power methods, such as single slope current starved DTC [9], are introduced at the DTC design level.

**Figure 5:**
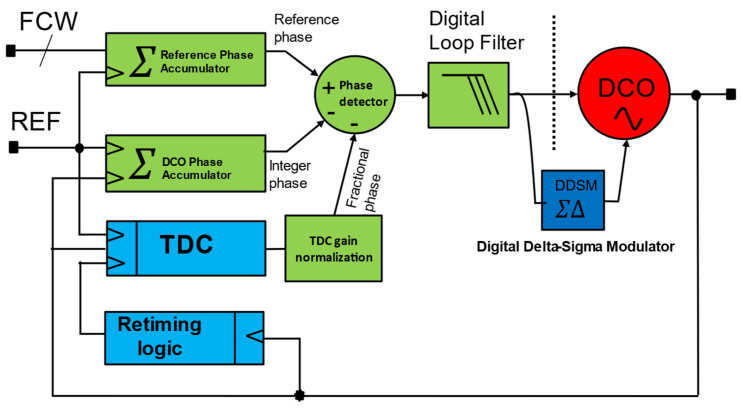
Typical accumulator-based ADPLL.

**Figure 6:**
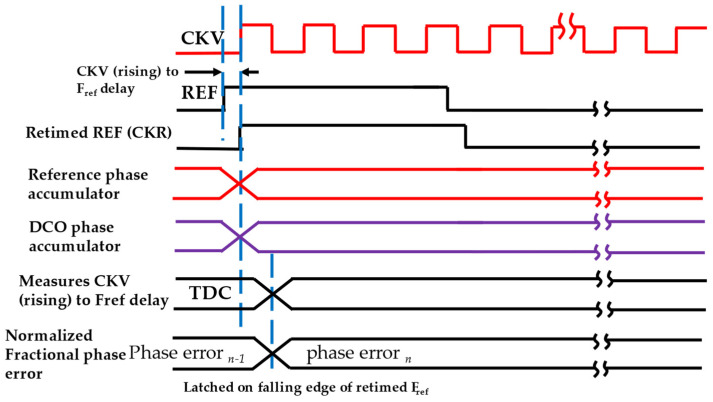
Typical timing diagram of accumulator-based ADPLL [32].

In some implementations, TDC is embedded into the DCO to save power and relax the TDC gain calibration requirement. An injection-locked ring-oscillator-based TDC is used in [34], which also complements the relaxation of TDC calibration, while some use embedded TDC-based ADPLL [11]. A typical accumulator-based ADPLL is shown in Figure 5. It uses a reference phase accumulator and a DCO phase accumulator. In a multi-clock domain digital system, all-digital sub-systems must be synchronized or operated in a master clock [6]. This is necessary to meet timing requirements and avoid metastability. Figure 6 shows the timing diagram [32] of the accumulator-based ADPLL. As shown in Figure 6, the DCO clock, which is also referred to as CKV, retimed reference (CKR) clock, and REF clock. The CKR (retimed) clock is used as the master clock for synchronization. TDC estimates the fractional phase between the DCO clock and the REF clock. The reference phase accumulator accumulates frequency command words (FCW) at every reference cycle, constituting the accumulating integer reference phase values. The TDC computes the time difference between the DCO edge and reference to compute the fractional phase error. The TDC works directly on the DCO clock, which leads to substantial power requirements. On the other hand, the DCO accumulator accumulates the DCO phase on every DCO cycle. However, to align the rates at which the phase quantities are compared, the DCO phase accumulator is sampled at the reference cycle, which is then compared by the phase detector to produce steady-state phase error. The computed steady phase error is going through DLF to produce a filtered version of OTW. In the closed loop operation, DLF can switch the ADPLL into type-II mode due to an additional digital integrator inside DLF. In addition to proportional and integral gain, DLF can also equipped with an infinite impulse response (IIR) filter to place higher-order poles to suppress the reference spur. The OTW, then drives the DCO towards the target after all it is negative feedback. The frequency of CKV can be given by the following expression.

(2) CKV=REF×FCW

#### 3.1.1. DTC Assisted Accumulator Based Low-Power ADPLLs

**Figure 7:**
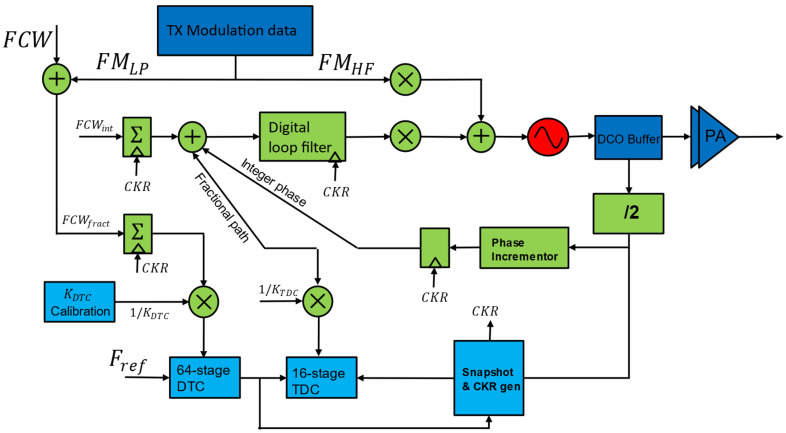
DTC-assisted accumulator type ADPLL [33].

As described, conventional accumulator-based ADPLL uses TDC, operated directly at the DCO (e.g., 2.4 GHz) clock, which directly burns a lot of power due to its high frequency of operation requirement. Hence, reducing the operating frequency has much potential to reduce TDC power consumption. Even though the TDC must cover one CKV period overall PVT conditions, it must cover significant phase errors during initial acquisition. One of the more practical ways to reduce the power consumption of TDC is to minimize the TDC range required with the help of DTC. This division of labor between the DTC and TDC allows for better resolution and accuracy in phase error measurement without requiring a high-resolution TDC. By reducing the range and complexity of the TDC, the architecture achieves significant power savings, making it ideal for low-power applications like IoT and WPAN devices. Both approaches are explored and implemented for ADPLL in [33], which is shown Figure 7. This approach exemplifies innovative architectural design can enhance the efficiency and performance of ADPLLs for low-power, high-precision applications [33]. This ADPLL is designed for WPAN applications, targeting standards like Bluetooth Smart and ZigBee in the 2.4 GHz ISM band and covers 2.4–2.7 GHz with a frequency accuracy of 60 kHz. A TDC is assisted with DTC to reduce the wide detection range and high resolution to decrease the power consumption. A DTC produces digitally controlled delay and can be achieved using a chain of digitally controlled delay cells. Each cell introduces a precise delay controlled by the digital input code that shifts the phase of one of the input signals (typically the reference clock) by a programmable amount.

The delay introduced by the DTC is computed based on the predicted phase error from the previous cycles. The phase prediction conducted by DTC effectively shifts the phase of the reference clock to coarsely align it with the DCO output before fine phase error detection by the TDC. After the DTC has coarse-aligned the phases, the residual phase error (typically a much smaller range) is measured by the TDC. Therefore, TDC operates at a lower detection range, requiring fewer bits for quantization, which reduces circuit complexity and power consumption. The DTC-assisted TDC is a key enabler for the ultra-low-power operation of the ADPLL, contributing to its total power consumption of 860 µW for 2.4 GHz applications. The DTC can be easily scaled to handle different frequency ranges, making the architecture versatile for various applications. Also, reducing the complexity of the TDC leads to a smaller overall circuit area. This ADPLL is implemented in a 40 nm CMOS process, leveraging digital architectures to minimize power consumption, marking a significant reduction. This ADPLL achieves a phase noise of −110 dBc/Hz at a 1 MHz offset, measured RMS jitter of 1.71 ps, and *FoM_J_* of −236 dB.

#### 3.1.2. Embedded TDC Based Accumulator Based ADPLL

**Figure 8:**
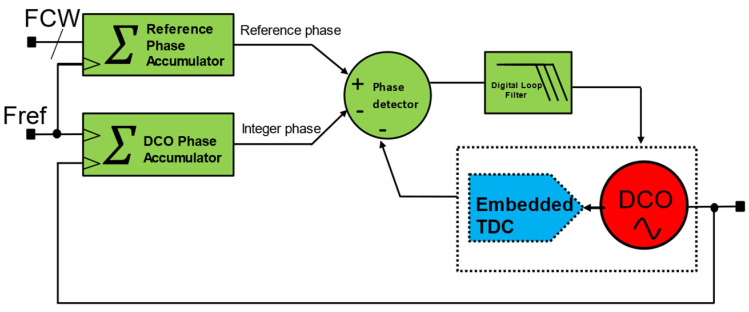
Embedded TDC-based accumulator type ADPLL.

Embedded TDC-based ADPLL can be realized in two ways. One way is to concurrently use the multiphase nature of multi-stage RO-based DCO as TDC and DCO. A simplified conceptual schematic diagram is shown in Figure 8.

The edges of RO-based DCO are captured at a reference rate to compute fractional phase error. The delay between two consecutive stages follows an RO-DCO period, eliminating the TDC normalization process to establish a PVT-independent TDC code. Depending on the power budget, the RO can be implemented in a differential or single-ended manner. However, differential implementation always increases the resolution of embedded TDC for a given stage and improves the supply noise and common mode noise immunity [11].

**Figure 9:**
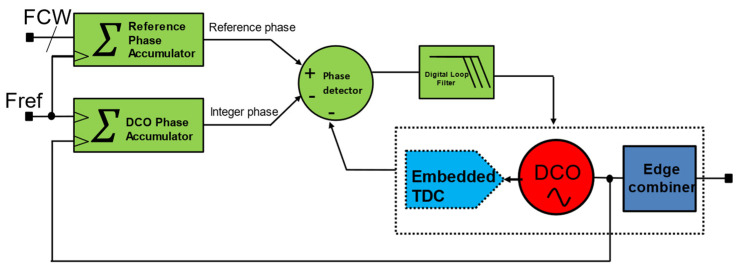
Embedded TDC-based accumulator type ADPLL with edge-combining.

Figure 9 shows an embedded TDC-based ADPLL, as reported in [11]. An all-digital ring oscillator (RO)-based Bluetooth low-energy (BLE) transmitter (TX) for ultra-low-power radios in short-range internet-of-things (IoT) applications was reported. The proposed ADPLL is used in a transmitter that offers wideband ADPLL using an embedded 5-bit TDC. This ADPLL also used a 4× frequency edge combiner to generate the 2.4-GHz signal. These techniques not only help reducing power consumption but also improve phase noise performance. Therefore, an efficiency-enhanced TX for short-range applications is achieved. The TX is prototyped in 40 nm CMOS, occupies an active area of 0.0166 mm^2^, and consumes 486 μW in its low-power mode while configured as a non-connectable advertiser [11]. The TX has been validated by wirelessly communicating beacon messages to a mobile phone.

#### 3.1.3. Accumulator-Based Low-Power ADPLL Using Injection-Locked RO-TDC

The embedded TDC concept proposed in [11] to utilize the internal phases of a ring-based multiphase DCO is one of the key concepts to achieve low-power accumulator-based ADPLL. However, achieving high-performance phase noise or lower RMS jitter is difficult from RO-DCO-based ADPLL due to the limitation on phase noise performance of DCO.

**Figure 10:**
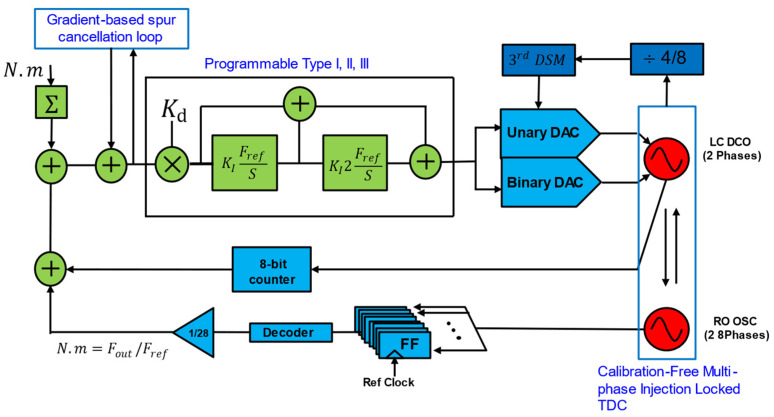
Injection-locked RO-based embedded TDC accumulator type ADPLL [34].

LC-DCO is preferred over RO-based DCO to overcome this difficulty due to its superior phase noise performance. Unfortunately, a single-stage LC DCO provides two complementary phases, which may not be sufficient to meet the stringent TDC resolution for making a functional ADPLL. Also, cascading multiple LC-DCO to form a ring is not feasible while considering area requirements, and the embedded TDC concept is impractical in this case. In this case, one viable option is to use a combination of LC-DCO and ring-based RO to form an embedded TDC-based ADPLL, as reported in [34], is shown in Figure 10. In this implementation, LC-DCO acts as a core DCO and ring-based RO acts as an injection-locked TDC (IL-TDC) for LC-DCO. In this way, the phase of RO aligned to that of DCO, and the delay between each stage (step-size of TDC) will be tracking the period of DCO, establishing inherent natural tracking of DCO period over PVT, preventing the need for TDC calibration. This ADPLL is implemented in 65 nm CMOS, occupying an active area of 0.027 mm^2^. The RO-TDC utilizes an interpolation flip-flop to improve the timing resolution DCO provides. The ADPLL achieves fractional-N operation without a multi-modulus feedback divider, avoiding its complexity and quantization noise. To improve the TDC linearity, a mismatch filtering technique incorporating a cross-coupled resistor network is proposed [34] to achieve a DNL less than 0.04 LSB of the TDC quantization level. The prototype consumes 3.2 mW with an operation frequency ranging from 600 to 800 MHz. The measured DPLL output phase noise at 800 MHz frequency achieves and −93 and −98 dBc/Hz at 1 kHz and 1 MHz offset, respectively [34].

Another implementation was reported in [29] using the dedicated RO, injection-locked to an LC-DCO. The resolution of the RO-based TDC depends on the number of stages in RO. In this case, a unit delay of TDC will follow the period of DCO, which eliminates the need for TDC normalization, and TDC operation is partially decoupled from DCO. Therefore, DCO can enjoy another degree of freedom to design independently to achieve better phase noise and power efficiency. This design used six-stage cross-coupled RO producing to form a 12-bit TDC, which is injection locked to the LC-DCO. The LC-DCO comprises a coarse capacitor bank, medium capacitor bank, and fine capacitor bank using MOM caps as it is less sensitive to PVT variations. In the coarse mode, the unit capacitor of the coarse capacitor bank is set to 25-fF to achieve a 14 MHz tuning step and 6-bit binary control to achieve a 1 GHz tuning range. The medium cap bank uses a smaller unit capacitor of 3.3-fF to achieve a 2 MHz tuning step, and 6-bit binary control is also adopted to achieve a 120 MHz tuning range. The fine banks are 9 bits that can cover 16 MHz with a tuning step of 40 kHz, which needs a 23-aF capacitor unit. The LC-DCO consumes only 354 µW, while the total power is 1.4 mW [29] during steady-state operation. This ADPLL is implemented in a 40 nm CMOS process covering 2.4–2.48 GHz. This ADPLL achieves −114 dBc/Hz @1 MHz and −221.1 dB *FoM_J_*.

### 3.2. Sub-Sampling Low-Power ADPLLs

In general, divider-based ADPLLs samples directly and compare the divided DCO clock (CKV) at every reference cycle with the help of TDC. Because of this sampling and comparison operation, this ADPLL can also be called a sampling PLL. Here, the phase comparison is done by TDC, which consumes much power due to the range and linearity requirement. A DTC can shrink the required TDC range to lower power consumption. There are several reported low-power ADPLLs in this category. However, the feedback MMD divider introduces quantization noise in the loop and limits the in-band noise. Because TDC noise is multiplied by N^2^ [35] due to the feedback being divided by N, this ADPLL offers multiple benefits, such as an extensive tuning range, PVT robustness, and fractional-N operation.

**Figure 11:**
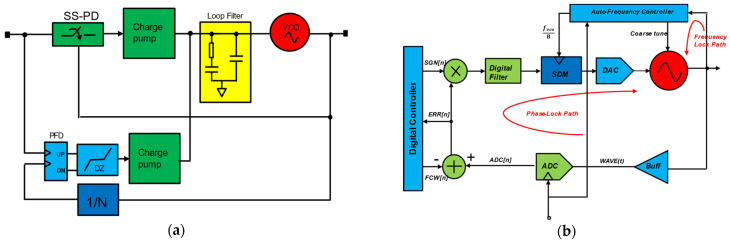
Sub-sampling PLL: (**a**) subsampling analog-PLL; (**b**) all digital sub-sampling PLL.

Whereas sub-sampling PLLs perform the divider-less operation by sampling oscillators directly by a sampling phase detector at the reference frequency. Due to the high gain nature of the sampling phase detection process, ideally, zero crossings of oscillator and reference should align, achieving frequency multiplication by N (target frequency ratio). The divider-less architecture removes the power consumption of the divider and N^2^ noise contribution [36]. The sub-sampling PLLs can be implemented in classical analog, as shown in Figure 11a, and one of the all-digital sub-sampling PLLs (SS-ADPLL) reported in [37] is shown in Figure 11b.

Since the DCO phase is sampled directly and compared using a sampling phase detector by the sub-sampling operation, this ADPLL is affected by frequency disturbances due to the multiple narrow-frequency lock-in ranges. This problem is prevented using a frequency-locked loop (FLL), which works with the out-of-dead zone (ODZ) detector. To save power, this FLL can be duty-cycled FLL (DC-FLL). The SS-ADPLL reported in [23] is fabricated in 40 nm CMOS and achieves phase noise performance of −109 dBc/Hz at 1 MHz offset while consuming 1.19 mW power. An analog SS-PLL is reported in [31] and is implemented at 40 nm using a 0.4 V supply. This SS-PLL exhibits a 236.6 fs rms integrated jitter, a −253.8 dB jitter-power figure-of-merit (*FoM_J_*), and a −76.1 dBc reference spur.

Another hybrid ADPLL approach uses an MMD-based sampling path for robust frequency acquisition and offers the best phase noise performance by switching to a sub-sampling path, as reported in [7]. The switching feedback leads to a 48% power consumption reduction from the DPLL, excluding the DCO power consumption. A proposed transformer-based stacked-gm ultra-low-power DCO consumes only 107 μW and gives −107 dBc/Hz at a 1-MHz offset frequency. A truncated constant-slope DTC (CS-DTC) is proposed to improve the power efficiency of the conventional architecture while retaining excellent linearity to achieve low fractional spurs. The presented fractional-N DPLL consumes 26 μW while achieving a jitter of 2.8 ps and a worst-case in-band fractional spur of −52 dBc.

### 3.3. Injection-Locking Low-Power ADPLLs

**Figure 12:**
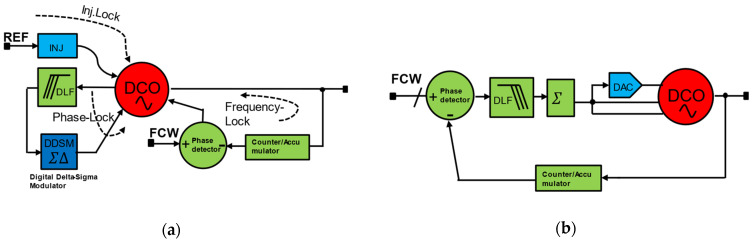
All-digital ADFLL: (**a**) all-digital FLL with DCO; (**b**) all-digital ADFLL with DCO and DAC.

Injection locking is another divider-less low-power technique to realize low-power synthesizers. In this class of ADPLLs, hardly any analog component except DCO is used directly inside the PLL. All state machines and control circuits are realized digitally; hence, it can be categorized as an all-digital implementation. One of the examples in this category is low-power all-digital frequency-locked loop (ADFLL) synthesizer for LO generation for RF receivers. A DCO or VCO can be injection-locked to an integer multiple of reference frequency when the DCO is tuned to be very close to the target frequency within a few KHz accuracy. A sophisticated digital algorithm can be implemented to lock the DCO to the reference by continuously tracking the DCO frequency in the event the DCO goes out of lock or out of target. Figure 12a,b show examples of injection-locking FLLs [30]. One uses DCO directly, and the other uses VCO in conjunction with DAC, which effectively forms the DCO.

#### 3.3.1. Adaptive All-Digital Frequency-Locked Loop (AADFLL)

A regular ADFLL will suffer frequency instability due to process, voltage, and temperature (PVT) conditions, and the loop will undergo a frequent locking process, leading to a loss of communication packets. Those lost packets disrupt transmission and necessitate retransmission, leading to poor system efficiency. To circumvent the above issues, reference [30] reports an adaptive all-digital frequency-locked loop (AADFLL), which proposes a specialized frequency control system designed to adjust its behavior for precise and power-efficient dynamic operation. This adaptive ADFLL incorporates mechanisms to adjust its parameters and functionality based on operating conditions, such as PVT variations or changes in the controlled oscillator’s characteristics. The AADFLL employs a binary search algorithm to adjust the digitally controlled oscillator (DCO) frequency during the locking process. This approach allows the loop to adaptively narrow the frequency error with minimal computational and power overhead. The AADFLL also used DCO gain estimation to enable the system to predict frequency error and update the loop parameters, such as loop gain and bandwidth, to balance power consumption and locking speed. This ensures accurate compensation for PVT variations, which can lead to frequency errors or instability and require manual calibration. The loop adaptively modifies its bandwidth and response based on the frequency error magnitude. A significant frequency error initiates faster correction, while more minor errors are handled with finer resolution for precise locking. This ADAFLL uses a low-dropout voltage regulator, which ensures a constant current supply to the DCO to minimize frequency drift due to current variation, maintaining stable operation even under varying supply conditions. This adaptive power regulation minimizes energy consumption, enhances the overall loop efficiency, and improves frequency accuracy. It continuously refines its frequency tracking to ensure the output remains locked to the desired reference frequency and provides robustness in response to real-time conditions [30]. This is suitable for power-constrained applications like Bluetooth low-energy (BLE) transceivers in IoT systems and other low-power transceivers. The design is fabricated and integrated into TSMC 55 nm CMOS technology for the BLE transceiver. The digital portion of the design is fully synthesizable, and its area is 1800 μm^2^ with a 1.233 K gate count [30]. The DCO draws 480 μA current from 0.55 V supply voltage at the center frequency. It has a frequency resolution of 4.8 kHz. The oscillator PN, at 1-MHz offset frequency from 2.44 GHz carrier frequency, is −122.85 dBc/Hz.

#### 3.3.2. Injection-Locked Frequency Multiplier (ILFM) Based Low-Power ADPLL

An injection locking-based frequency multiplication is yet another low-power method of frequency synthesis. This eliminates the need for a TDC, and DTC simplifies the architecture and reduces power consumption. This ADPLL provides low complexity and low-cost design with reduced area requirements. RO-based DCO or LC-based DCO can be locked to harmonics of reference frequency to achieve integer frequency multiplication with a high phase coherence between the injected signal and DCO. If the free running frequency of such DCO is close to the target frequency, DCO will be locked to the injected reference frequency, exhibiting superior phase noise performance. However, tuning DCO frequency towards a target within a few KHz accuracy would require FLL, and that DCO would require a very fine discrete tuning step. A 2.4 GHz ultra-low-power all-digital phase-locked loop (ADPLL) featuring an injection-locked frequency multiplier (ILFM) and continuous frequency tracking loop (CFTL) for low-power IoT applications is reported in [21]. The system operates in a closed-loop configuration to achieve and maintain frequency locking to the reference signal injected into the ILFM, which generates the initial high-frequency signal. The CFTL compares the output frequency with the reference and adjusts the DCO control to minimize frequency error. The DCO generates the final stable output frequency, locked to the reference. This CTFL uses a binary search algorithm for fine-tuning the DCO output frequency, dynamically adjusts the DCO frequency to compensate for PVT variations, and operates continuously in the background, ensuring frequency stability and minimizing frequency drift after reference injection. This design uses ultra-low-power LC-DCO) designed for high efficiency and low power, consuming only 0.46 mW and operating in the sub-threshold region with a supply voltage of 0.5 V. The DCO includes three capacitor banks (MSB, LSB, and Fine) for coarse and fine frequency tuning, achieving resolutions of 18.7 MHz, 72 kHz, and 4.8 kHz, respectively. This ADPLL works for the range from 2.402 GHz to 2.480 GHz and achieves spot phase noise −111.15 dBc/Hz at 1 MHz offset from the carrier frequency and achieves an integrated jitter of 1.652 ps. The active area of the ADPLL is 0.129 mm^2^. The fully digital implementation minimizes circuit complexity and silicon area. The combination of ILFM and DCO achieves low phase noise and jitter, which is essential for RF applications like IoT and BLE transceivers. This system efficiently combines frequency synthesis and stabilization techniques to meet the demands of modern energy-constrained ultra-low-power wireless communication systems.

## 4. Low-Power DCOs Using Transformer Coupling for ADPLLs

Throughout the discussion, it is established that the TDC power consumption can be minimized using several techniques as reported, such as DTC-assisted TDC, embedded TDC, and injection locking TDC. Then, the following key block to optimize is DCO, which is the highest power-consuming block. There are later references which utilize transformer coupling to improve the power efficiency of DCOs. A transformer coupling for quality (Q) factor enhancement of the DCO tank to improve signal-to-noise (SNR) of oscillator amplitude and thereby improve the phase noise is implemented in [9]. Reference [12] uses transformer coupling to create multiple modes of oscillation (e.g., fundamental and second harmonic) to suppress flicker noise up-conversion to improve phase noise performance for the fundamental mode.

### 4.1. Inverse Class-F DCO-Based Accumulator Type ADPLL Using a Transformer

A 529 µW fractional-N all-digital phase-locked loop (ADPLL), designed for Bluetooth low-energy (BLE) and IoT applications, fabricated in 65 nm CMOS [12]. It achieves ultra-low-power consumption and robust performance, leveraging innovative features such as combined vernier and flash TDCs. This new TDC design extends the input range and enables background gain calibration, maintaining stability against PVT variations [12]. Also, this design ensures fast lock time and better stability.

**Figure 13:**
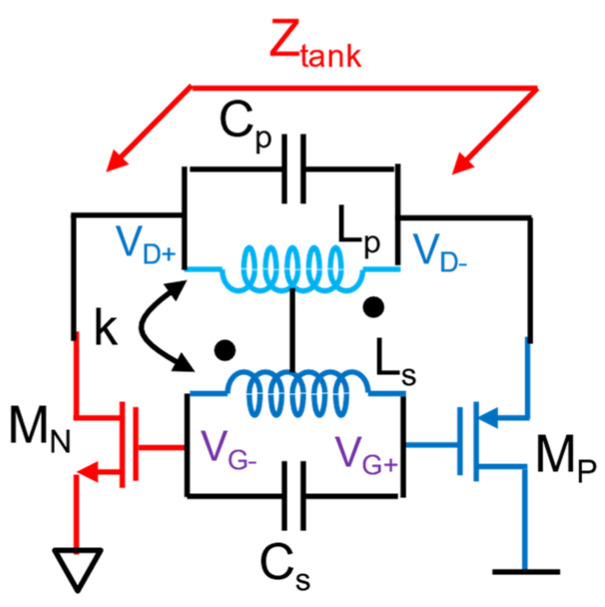
Simplified inverse class-F DCO [12].

**Figure 14:**
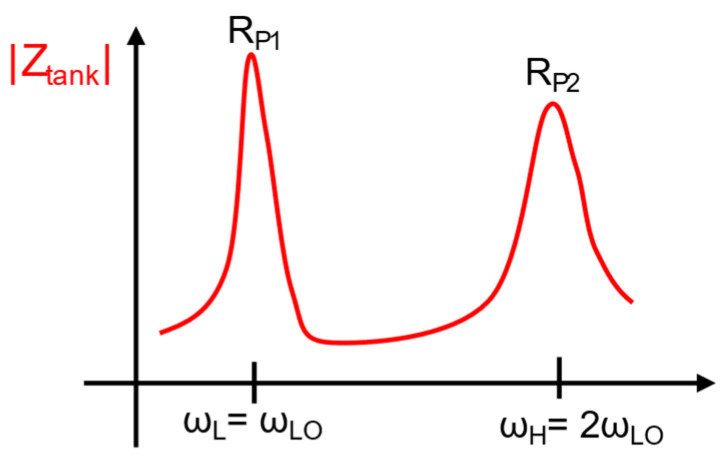
DCO tank impedance showing the first and second harmonics [12].

This design also features inverse class-F DCO with a tightly coupled primary transformer with optimal coupling co-efficient k = 0.6, tuned to fundamental and second harmonic frequency. Figure 13 shows the simplified schematic of inverse class-F DCO, and Figure 14 shows the representative impedance vs. frequency plot. The frequency alignment avoids the need for independent tuning for fundamental and second-harmonic using multiple capacitor banks, reducing the complexity. Coarse frequency tuning is implemented on both primary and secondary coils. Fine-tuning and delta-sigma modulation (ΔΣ) are confined to the secondary coil, simplifying the layout, and maintaining performance.

Conventional class-F oscillators require complex 2D capacitor tuning for harmonic alignment, which is challenging to automate and prone to PVT variations. The class-F^−1^ DCO eliminates this by leveraging the transformer tank’s natural harmonic alignment; i.e., the fundamental frequency $\omega_{L}$ and 2nd-harmonic frequency $\omega_{H}$ of the oscillator are aligned, which can effectively suppress the noise contribution from the negative $g_{m}$ transistors. According to [12], $\omega_{L}$ and $\omega_{H}$ can be expressed as

(3) $$\begin{array}{ll} \omega_{L,H}^{2} & =\frac{1+\xi \mp \sqrt{(1+\xi {)}^{2}-4\xi \left(1-k^{2}\right)}}{2\left(1-k^{2}\right)}\cdot \omega_{2}^{2}\\ & =\Omega_{L,H}^{2}(\xi ,k)\cdot \omega_{2}^{2} \end{array}$$

where k is the coupling coefficient, $\xi =L_{S}C_{S}/L_{P}C_{P}$, and $\omega_{2}=1/\sqrt{L_{S}C_{S}}$. To guarantee $\omega_{H}/\omega_{L}=2$, the transformer tank needs to satisfy the following expression [12].

(4) $$16\xi^{2}+\left(100k^{2}-68\right)\xi +16=0$$

The tightly coupled transformer tank demonstrates the high-Q impedance peaks at $\omega_{LO}$ and $2\omega_{LO}$ can be obtained by choosing a small k of 0.38 and a large ξ of 3, which enlarges $2^{\mathrm{nd}\;}$-harmonic voltage and extends the flat span where the impulse sensitivity function (ISF) is minimum. Together with a high voltage gain from $V_{D}$ to $V_{G}$ provided by the transformer, this tight transformer coupling also saves die area compared to loosely coupled designs. Simplified switched-capacitor banks reduce layout complexity. Capacitance mismatches and PVT variations have minimal impact on performance due to the balanced transformer design. This ADPLL achieves 868 fs jitter in fractional-N mode, 684 fs in integer-N mode, and *FoM_J_* of −244 dB in fractional-N mode and covers BLE channels from 2400–2480 MHz [12]. The ADPLL demonstrates a competitive solution for ultra-low-power frequency synthesis, setting benchmarks in jitter, power, and spur suppression, and optimized for BLE and IoT devices, emphasizing energy efficiency for battery-powered and energy-harvesting systems.

### 4.2. ADPLL Using Transformer-Based Stacked Gm Resonator for DCO

**Figure 15:**
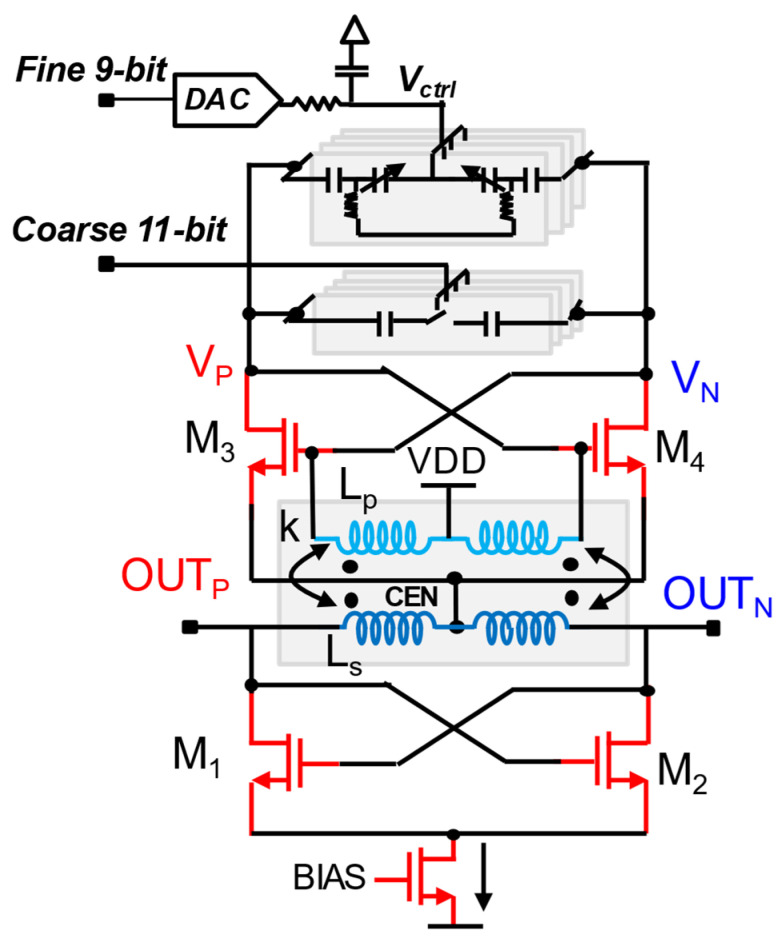
Transformer coupled stacked *gm* DCO [9].

Figure 15 shows the DCO design uses stacked-gm architecture and incorporates stacked transconductance (gm) stages, where multiple NMOS transistors share the same bias current. Stacking gm devices will increase the effective gm for the given bias current. Therefore, DCO current can be maintained above critical current to avoid failures in DCO and divider. This helps DCO to achieve more output signal power than noise power without requiring increased current, leading to improved oscillation amplitude. The oscillator also employs a transformer-based tank to enhance the resonant circuit’s quality factor (Q). Unlike designs with natural harmonic alignment (e.g., inverse class-F), this DCO focuses on generating a strong fundamental oscillation without explicitly targeting the suppression of harmonics through resonance. So, with the lesser current for higher effective gm and high-Q tank, the phase noise can be reduced by minimizing the flicker noise up-conversion to significantly improve phase noise performance compared to traditional LC tank designs. The transformer-based approach also reduces the need for large capacitors or inductors, saving die area [9]. The design demonstrates resilience to process, voltage, and temperature (PVT) variations, ensuring consistent performance in real-world conditions.

Fine and coarse frequency tuning is achieved using binary-weighted capacitor arrays for precision control. Delta-sigma modulation (ΔΣ) may be employed for finer resolution, ensuring linear and stable frequency adjustments. The DCO consumes 107 µW, contributing significantly to the overall low-power operation of the PLL. This ADPLL implements a hybrid approach combining a classical divider-assisted ADPLL-based sampling path and sub-sampling path using SS-ADPLL. A unique switching method between these paths to enable high-performance and power-efficient design is ideal for battery-powered and energy-harvesting IoT applications. More discussion on hybrid ADPLL will be held in the coming subsections. This DCO design also achieves a wide tuning frequency range from 2.1 GHz to 3.1 GHz, enabling compatibility with various wireless standards such as Bluetooth low-energy (BLE) and ZigBe, and offers an effective solution for IoT and wireless communication applications.

## 5. Low-Power Hybrid ADPLLs

As the name implies, hybrid PLL combines two types of PLLs connected to achieve overall system goals. These two PLLs can be connected in parallel or series. The system goal could be meeting specific power numbers, phase noise performance, area, lock-time, etc. There are few reported examples in the process of making hybridization; i.e., two PLLs can be formed of two analog PLLs or two digital PLLs and a combination of analog and digital PLLs.

### 5.1. Cascaded Hybrid Low-Power ADPLL (Master-Slave PLL)

**Figure 16:**
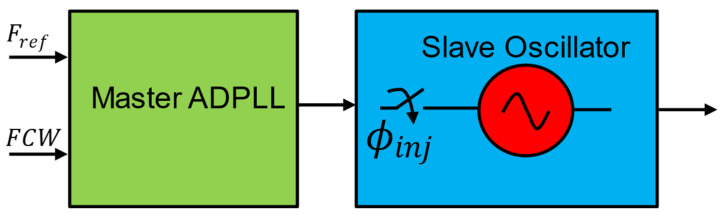
Master-slave (cascaded) injection-locked ADPLL.

Another hybrid approach described in [7] cascades two PLLs to reap the benefit of injection locking to generate high-frequency multiplication with good phase coherence. Figure 16 shows the simplified block diagram of cascaded hybrid ADPLL. This work cascades two PLLs in master-slave mode, aiming to generate high-frequency synthesis with extremely low power.

The architecture utilizes a master-slave design to achieve low power consumption and fast frequency locking. The master PLL works on a 9 MHz reference and generates 75 MHz at extremely low power. Then, this 75 MHz is used as an injection locking signal to lock the slave PLL’s DCO and generate a low-phase noise signal whose frequency multiplied 32 times. Injection-locked oscillator (IL-OSC) is used to produce the final 2.4 GHz signal with four-phase outputs. This work presents a 250 µW 2.4 GHz fast-lock fractional-N phase-locked loop (PLL) designed for ultra-low-power (ULP) applications, such as wireless communication systems and implantable devices. A successive approximation register (SAR)-based fractional-N coarse-lock unit (CLU) enables rapid coarse frequency locking in the master PLL [7]. At the same time, a fine frequency initialization unit (FIU) in the slave oscillator ensures the IL-OSC operates within its locking range by compensating for quantization errors. The PLL achieves fast locking through two modes: start-up locking in 22 µs and instantaneous relocking in 1 µs, making it ideal for power-sensitive applications requiring rapid transitions. The system is highly power-efficient, consuming only 250 µW, with the master PLL and IL-OSC consuming 88 µW and 162 µW, respectively. An error compensation circuit in the IL-OSC adjusts the free-running frequency in real time, mitigating PVT variations and minimizing phase skews. The PLL demonstrates a phase noise of −106.5 dBc/Hz at a 1 MHz offset, an integrated jitter of 1.652 ps, and a frequency range of 2.31 GHz to 2.55 GHz, achieving a figure of merit (FoM) of 0.102 mW/GHz [7]. Fabricated in 65 nm CMOS with a compact 0.083 mm^2^ chip area, this PLL offers a power-efficient and robust solution for IoT and BLE applications.

### 5.2. Dual Loop Hybrid Low-Power ADPLL

Hybrid PLLs often use dual-loop (parallel-loops) to achieve simultaneous lock-time and phase noise performance. For instance, a PLL employing SS-ADPLL could suffer mediocre lock-time. This is because the DCO of SS-ADPLL could lock to any of the integer-N multiples of REF other than the target. In this case, FLL is required as an auxiliary loop that shuts off once the lock is acquired. However, these techniques could still suffer due to poor lock time [9]. In this case, an accumulator-based ADPLL or divider-assisted ADPLL could aid the loop-locking process and can eventually be shut off. This ensures that one loop will be active at a time, providing sufficient phase noise for specific applications requiring high phase noise.

#### 5.2.1. Switchable Dual-Loop Hybrid ADPLL

**Figure 17:**
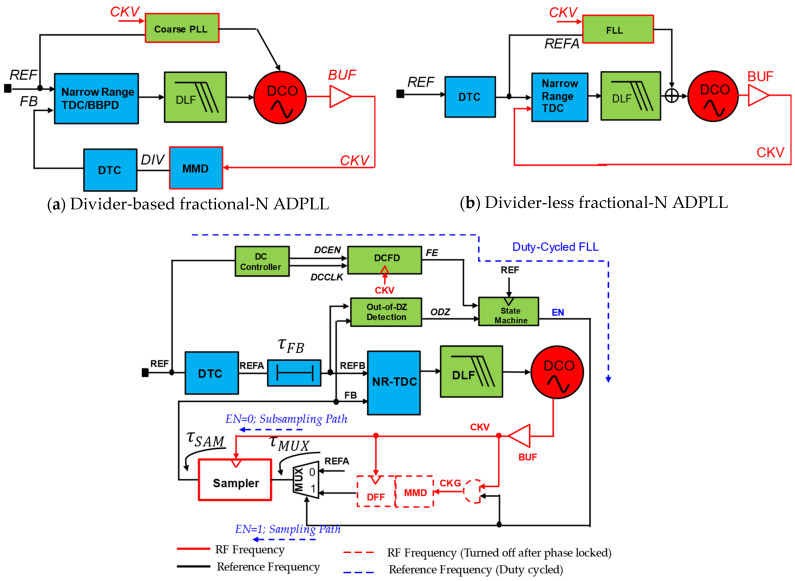
Hybrid ADPLL combining divider-based ADPLL (**a**) and SS-ADPLL (**b**) [9].

A high-performance, low-power ADPLL is demonstrated in [9]. This implementation demonstrates a high-power efficient DCO with a transformer coupled with stacked gm, which is discussed in the previous section. Another notable architectural choice was combining divider-assisted ADPLL and SS-ADPLL and selecting between these loops [9]. In this case, divider-assisted ADPLL could aid the loop-locking process and can eventually be shut off. Figure 17 shows two key ADPLLs. One is classical divider-based ADPLL shown in Figure 17a, which uses a multi-modulus divider (MMD) in the feedback path. This can be called sampling PLL, as a phase of MMD is sampled and compared at the reference rate. Also, narrow-range TDC or BBPD can be used for phase quantization. This narrow-range TDC will consume less power with the help of DTC. However, power consumption due to MMD can further be eliminated if ADPLL takes in another divider-less architecture, as shown in Figure 17b. This ADPLL can also employ a narrow-range DTC-assisted TDC as a phase detector, as shown. As this ADPLL samples the DCO phase directly, this is sub-sampling ADPLL. This approach promises very low-power operation. However, due to its sub-sampling nature, this ADPLL can lock DCO to the multiple narrower lock range. So, to incorporate a robust lock to the right target frequency, this ADPLL works with an out-of-dead zone (ODZ) detector and duty-cycled FLL (DC-FLL). This DC-FLL improves power consumption and eliminates the need for MMD and excess noise in the loop. Nevertheless, sampling ADPLL with MMD can improve the frequency range, locking process, and calibration. The benefits of both architectures are combined to form a hybrid ADPLL, as shown in Figure 17. Thus, this ADPLL can seamlessly switch between dual-loops employing sampling mode and sub-sampling mode, achieving an extremely low power of 265 μW [9].

This approach removes the power consumption of the MMD. It offers the best phase noise performance, enabling a sub-sampling path while achieving robust frequency acquisition characteristics when frequency disturbances occur by activating the sampling path. The switching feedback leads to a 48% power consumption reduction from the ADPLL, excluding the DCO power consumption. A proposed transformer-based stacked-gm-based DCO [9] consumes only 107 μW and gives −107 dBc/Hz at a 1-MHz offset frequency. A truncated constant-slope DTC (CS-DTC) is proposed [9] to improve the power efficiency of the conventional architecture while retaining excellent linearity to achieve low fractional spurs. The presented fractional-N ADPLL consumes only 265 μW while achieving a jitter of 2.8 ps and a worst-case in-band fractional spur of −52 dBc. The obtained power consumption is nearly 2.5 times smaller than that of a state-of-the-art low-power fractional-N ADPLL [9].

#### 5.2.2. CP-PLL Based Dual-Loop Hybrid ADPLL

The CP-PLL offers the best phase noise performance, while the accumulator-based ADPLL offers faster lock-time. Therefore, combining those two PLLs could achieve the best phase noise and faster lock-time performance. Such a hybrid-PLL is proposed in [8] implemented in a 7 nm FinFET CMOS that combines the best advantages of ADPLL and CP-PLL. The proposed PLL also introduces a periodical phase realignment via the reference clock and ultrashort pulse for resetting the phase detector (PD). The hybrid PLL covers 0.2–4 GHz and settles in 0.6 µs. It emits low −52 dB reference spurs in the conventional mode, and 1.05 ps and 0.62 ps integrated jitter in the conventional and realignment modes, respectively.

## 6. Conclusions

This paper reviewed various ADPLL architectures for ultra-low-power implementation in detail and briefed on the necessity of ADPLL for the completeness of the discussion. This review starts by capturing a hierarchical tree of low-power ADPLLs by compiling the data from the literature. More focus is devoted to divider-less ADPLLs as there is significant potential for low-power implementation, and numerous works in the literature have reported remarkable high-performance ADPLLs. Table 2 compares different ADPLLs in terms of features, power consumption, and challenges. This review also captures the basics of divider-assisted ADPLL and BB-ADPLL for completeness.

Divider-less ADPLLs eliminate the need for MMD, which helps minimize power consumption. In accumulator-based/counter-based ADPLLs, TDC, which works on a DCO clock, burns enormous amounts of power owing to its high frequency of operation. A DTC-assisted TDC helps to minimize power consumption in two ways. One way is to reduce the range requirement, reducing power as fewer components are used than a stand-alone TDC [11,12,15]. Another way is to reduce the sampling rate or the rate at which the TDC is operated for eventual reduction in power consumption. Even though the DTC-assisted TDC works for low-power, accurate DTC gain calibration is required to limit the amount of reference spur at the ADPLL output [33].

Then, the review investigated other divider-less architectures, such as injection-locking ADPLL [21] and ADAFLL [30], demonstrating extremely low power consumption and performance. As can be seen, both ADPLL and ADAFLL also do not require MMD, potentially significantly reducing power consumption. However, the DCO design for injection-locking ADPLL requires a finer frequency step size, for example, 4.8 KHz, as reported in [21], making it challenging to achieve frequency range and phase noise performance simultaneously. The fine frequency step size is required to ensure that DCO frequency can be driven very close to N times the injected signal to obtain good phase noise from the DCO. Where N is the target frequency multiplication factor, however, this architecture has a drawback due to the inability to generate fraction-N frequency. A fractional-N injection-locking RO is reported in [35] using a novel technique.

The SS-ADPLL also does not require MMD in the feedback, potentially reducing overall phase noise contribution. A low-power implementation is reported in [23], while achieving good phase noise performance and reference; also, low-power performance is reported in [9] during the sub-sampling mode of operation. The sub-sampling architecture eliminated the N^2^ factor noise contribution due to MMD, as in the case of divider-based ADPLLs [36]. Also, with the help of DTC assistance, sub-sampling ADPLLs can synthesize fractional-N frequencies. However, due to the sub-sampling nature, the DCO used in this ADPLL could lock to multiple narrow lock ranges. Therefore, PVT variations require a carefully designed auxiliary FLL loop to re-lock the PLL.

This review also exemplifies two key state-of-the-art low-power DCO implementations reported. One is class-F^−1^ DCO using transformer coupling [12], and the other DCO uses stacked *gm* DCO using transformer coupling [9]. This transformer coupling improves the tunable capacitor-bank layout, reduces area, and provides a compact capacitor-bank. This review also focuses on hybrid ADPLL reported and compiles various level hybridizations, including the example of cascaded hybrid ADPLL featuring extremely low power [7]. Another low-power hybrid implementation utilizes a switchable loop between the sampling and sub-sampling paths [9]. Another hybrid ADPLL is discussed here, even though it is out of the category of sub-milli-watt ADPLL, to show that lock-time can be reduced significantly [8], and the reported lock-time was 600 ns.

## Figures and Tables

**Table 2:** Comparison of different ADPLL architectures: features, power consumption, and challenges.

ADPLL Type	Key Features	Power Consumption/Performance	Challenges
**Divider-Assisted ADPLL** [14,18,20]	Utilizes multi-modulus-divider (MMD) in the feedback path	Higher power consumption due to MMD	Increased complexity, power-hungry TDC
**Bang-Bang ADPLL (BB-ADPLL)** [10,16,17]	Uses a bang-bang phase-frequency detector (BB-PFD)	High power, but simple and robust for integer-N operation	High power in high-performance designs and requires DTC for fractional-N operation
**Accumulator/Counter-Based ADPLL** [11,12,15,24,25]	Eliminates the frequency dividers and operates in the true phase domain [6]	Low-power by eliminating MMD	TDC still consumes a significant portion of power; requires DTC assistance for very low-power implementation
**Injection-Locked ADPLL** [21]	Employs injection locking for phase synchronization to achieve good phase noise performance	Very low power; minimal circuitry	Limited to integer-N operation
**Adaptive All-Digital Frequency-Locked Loop (AADFLL)** [30]	Uses a frequency-locked loop instead of a phase-locked loop [30]	Very low power; minimal circuitry	Requires fine frequency control; limited fractional-N synthesis
**Hybrid ADPLL** [7,8,9]	Combines ADPLL with CP-PLL or other ADPLL architectures	Power consumption varies from high to very low, depending on the hybrid approach; optimized trade-offs can achieve ultra-low power and fast lock times	Complexity in design and switching between modes
**Sub-Sampling ADPLL (SS-ADPLL)** [23]	Direct phase comparison using the reference clock	Very low power; minimizes MMD impact leading to good phase noise performance	Sensitive to frequency disturbances; requires out-of-dead zone (ODZ) detection for frequency stability

## Data Availability

Data are contained within this article.
